# Barriers for Cataract Treatment among Elderly in Sri Lanka

**DOI:** 10.1155/2019/6262456

**Published:** 2019-03-03

**Authors:** Nilanga Nishad, S. A. Hewage, K. Arulmoly, M. S. Amaratunge, J. de Silva, K. T. A. A. Kasturiratne, P. K. Abeysundara, A. R. Wickramasinghe

**Affiliations:** ^1^Teaching Hospital, Batticaloa, Sri Lanka; ^2^Postgraduate Institute of Medicine, University of Colombo, Sri Lanka; ^3^Radiant Eye Hospital, Ja-ela, Sri Lanka; ^4^Teaching Hospital, Kandy, Sri Lanka; ^5^Department of Public Health, Faculty of Medicine, University of Kelaniya, Sri Lanka; ^6^North Colombo Teaching Hospital, Ragama, Sri Lanka

## Abstract

Cataract is still the leading cause of blindness. Many government institutes and voluntary organizations in Sri Lanka are providing free treatment services to patients with cataract. Still people are not patronizing the available free services; thus they have to live with impaired vision or blindness. This paper describes the barriers for cataract treatment among the population over 60 years of age. Out of 470 elders, 379 were found to have some kind of cataract. This study demonstrated lack of awareness and knowledge, socioeconomic factors, and misconceptions as the main barriers for cataract treatment which has led to a lower cataract surgery rate irrespective of the high cataract prevalence reported. Findings of this study highlight the importance of cataract as a common health problem in elderly and need for removal of the barriers for its treatment which should be given due prominence in the formulation of public health policy in Sri Lanka at the earliest.

## 1. Introduction

Cataract is still the leading cause of blindness in the world and principal cause of visual impairment globally (43% and 33 %, respectively) [1]. Its prevalence increases with age from less than 5% in persons fewer than 65 years of age to approximately 50% in those 75 years of age and older; thus it has become a problem associated with getting old [2]. Epidemiologic studies on risk factors for cataract over the last few decades have identified age-related cataract as a multifactorial disease [3].

Poor visual function was associated with limitations in mobility, activities of daily living, and physical performance [4]. Blindness and visual impairment are not only health issues for the elderly but also significant determinants for all aspects of life, including the quality of their lives [5].

The cataract surgical rates (CSR), defined as the number of cataract surgeries done per million of the population per year, has significantly increased over the last decade in Sri Lanka [6]. However, when compared with the prevalence of cataract blindness, it is obvious that the majority of patients are still without appropriate ophthalmologic services [7]. Many government institutes and voluntary organizations in Sri Lanka are providing free treatment services to patients with cataract. Many people are still not patronizing the available free services; thus they have to live with impaired vision or blindness.

Negative aspects of treatment seeking behavior such as lack of financial support, commitments to the family, accessibility barriers, and lack of awareness on illness or on available treatments are the main reasons why people with visual impairment are not getting the treatment that they require in other Asian countries [8, 9].

A review done and published in 2007 in India identified financial reasons, distance, fear, lack of service awareness, lack of support, or other obligations as barriers for cataract treatment. Financial barriers were the major reason for not availing cataract surgery services [10].

Cost of treatment, being too old for surgery, inability of a family member to accompany the patient to a health care facility, lack of knowledge about cataract, and concerns about the quality of local services were identified as barriers for cataract in a study done in a rural area of China in 2008 [11].

This paper describes the barriers for cataract treatment among the population over 60 years of age in Mahara Divisional Secretariat area.

## 2. Methods

Gampaha district is adjacent to Colombo the capital of Sri Lanka. It has 13 divisional secretariat divisions (DS) and has a population of 2 294 641. The elderly population in Gampaha district according to census in 2012 March is 290386.

The target population was persons over 60 years of age living in Gampaha district. Those people who are critically ill and bed bound were excluded from the sampling frame. Eighteen Grama Niladhari (GN) divisions (DS divisions are divided into smaller clusters) were selected based on simple random sampling using computer generated random numbers without replacement. In the next level, 30 participants from each GN division were selected using the same technique without considering the size of elderly population in each GN division. Calculated sample size was 540 and 470 participated with 87% response rate.

An interviewer administered questionnaire was used to collect data from the participants. A data recording sheet was used to document the results of the eye examination. Principal investigator interviewed all participants. Data collection was carried out over 18 sessions. Slit lamp examination was carried out to diagnose cataract. Cataracts were classified as nuclear, cortical and subcapsular, and graded. Visual analog scale was used to collect data on attitudes and beliefs on cataract treatment.

Subjects diagnosed with cataract by slit lamp examination were again interviewed by the investigator with a self-administered questionnaire on assessing the barriers for treatment.

The participants positive for cataract were counseled for surgery and arranged the treatment on their will free of charge. Participants with other visual problems like diabetic retinopathy, age-related macular degeneration, and glaucoma were referred and participants who required spectacles were given them free of charge. Barriers were analyzed with percentages, percentiles, and 95% confidence intervals. Students t tests and chi-square tests were used to calculate the significances with 0.05 as the p-value.

Ethical clearance was obtained from the Ethical Review Committee of Faculty of Medicine, University of Kelaniya, Sri Lanka (Ref –P108/07/2012).

## 3. Results

Table 1 describes the basic demographic characteristics of the population in the sample. The sample consisted mainly of females as expected because the age expectation of females is more than males in the country. Other characteristics are described in the table.

Prevalence of any cataract of at least one eye including operated eye was 379 (80.6%) (95 % CI 76.8 to 84%) and 346 (73.6%) (95 % CI 69.5 to 77.4%) when operated eye is excluded (Table 2).

### 3.1. Barriers for Cataract Surgery (People with Cataract Were Only Included)

Many people with cataract were not aware that they have cataract and more than fifty percent people were not aware of lens implantation and the type of anaesthesia used in cataract surgery. Almost forty percent of people were not sure about weather cataract can be cured.

Nearly sixty percent of people thought that a person should restrict his or her routine activities for one or more months after surgery. Gender did not show any difference in knowledge on cataract or cataract status of individuals. People with ordinary level education (secondary education) and above knew that government hospitals are providing cataract treatment more than people with less than secondary education (p=0.02) (Table 3).

Most of the people were not able to bear the cost of the surgery (n=234, 67%) although 189 (54.3%) were willing to do so. Most of the cataract people were able to find transport facilities (n=311, 89%) and cost for drugs (n=280,80.7%) and 268 (77%) think they can get assistance from relatives and neighbors at and after surgery. (Table 4)

There were no gender differences in the financial and other logistics supply for cataract surgery or treatment, but males thought that they can have more help at the surgery than females thought (p=0.01).

Sixty-two (18%) people were postponing the cataract treatment at the moment and the commonest reasons were family commitment and their busy schedule (Table 5).

According to Table 6, thirty-five percent of people who had cataract had some fear for cataract surgery and 13.5% were very much afraid of the surgery. Females had a significant amount of fear (p=0.03). Seventy-five percent of people had some worry about the cost they have to incur and 33% were very much worried about it. Lower educational status (p=0.01) and lower income level (P=0.01) were significantly associated with it. Twenty percent of people were much worried about worsening the current vision after a cataract surgery. Twenty-two percent of people thought they can function with one eye. Forty-four percent thought that they have to limit their routine activities and they were afraid of treatment due to that reason and 13.5% had a strong fear on it. Lower educated group (p=0.01) and females (p=0.04) significantly had that fear. Thirty-four percent of people thought that there may not be intervention for the natural process of reduced vision and 6.3% firmly believed in it. Lower educational status was significantly associated with it (p=0.01). Forty-eight percent thought that there would be suffering for their loved ones and family due to their surgery and 13% of people firmly thought of it and lower educational status was associated with it (p=0.03). Sixty-five percent of people thought that they have to wait long periods in waiting lists and hang around in hospital clinics for long periods and 25% were thinking of it strongly. Currently married group was associated with that thought (p<0.02). Thirty-five percent of elders thought that there was no use even if they got a better vision as they are older now and 12% had the firm belief on it. Lower income (p=0.02), lower educational status (p=0.01), and higher age (p=0.03) were associated with it. Further details on the attitudes and beliefs are shown in Table 6.

Analysis of variances was carried out using age as a continuous variable and other four as categorical for all 11 statements and income was significantly associated (p=0.016) with “I am afraid to undergo cataract surgery.” Age was associated with “I believe that I could manage my work with one eye” (p= 0.023), “I am afraid that I will have to wait long periods of time in the waiting list to do the operation” (p=0.001), and “I believe that I am too old to undergo an eye surgery” (p= 0.028). Marital status was associated with “I am afraid that I will have to wait long periods of time in the waiting list to do the operation” (p= 0.012).

The common reasons they highlighted not getting the treatment was the financial reasons, fear of surgery, and various myths regarding the procedure and side effects.

## 4. Discussion

This study demonstrated lack of awareness and knowledge, socioeconomic factors, and misconceptions as the main barriers for cataract treatment which has led to a lower cataract surgery rate irrespective of the high cataract prevalence reported. Most of the people were not aware that they had cataract. Awareness of treatment procedures and the fact that cataract can be cured was poor. Misconceptions on surgery and postoperative procedures may also prevent people from getting treatment. Financial reasons were the commonest barrier reported by people for not seeking treatment. Educational level, income, age, and gender were associated with poor attitudes and false beliefs on several aspects of treatment seeking behavior. Fear of the surgery, worry about the cost, thinking that vision in one eye is adequate for daily activities, fear of limitation of daily activities after surgery, burden on family members, fear of waiting in long queues and waiting lists, and not having a desire to improve vision were other barriers that were identified in this study. One observation in study participation which was highlighted but may not have a major impact is the male to female ratio which is not compatible with census data. The female to male ratio in our sample is just above 2, but in Gampaha district it is more than 1.5 but not above 2.

Seventy-four percent of the people with cataract were not aware that they have cataract. Knowledge on cataract as a disease and available treatment options and curability was poor among the elderly population in this study. Forty-seven percent of people were not aware that surgery is the mainstay of treatment of cataract and it is a major concern that most of the public health should pay attention to. Accepting blindness as a consequence of elderly may be a cause for this and people with early stages may not be aware of the little changes in vision. Lau et al. (2002) in Hong Kong reported this to be about 40% in their population based study [12]. Fifty-one percent of cataract patients were not aware that there is a need to implant a lens as treatment and 70% of them were not aware of the type of anaesthesia they would receive during surgery. Desai and Copeland (2013) also described low awareness as one of the barriers for cataract surgeries in USA. Thirty percent of cataract patients in this study were not aware that government institutions provide treatment facilities and 25% were not aware that such services are available in the private sector [13]. Boudville et al. (2012) carried out a qualitative study on access to cataract surgery among the indigenous population in Australia and reported that low awareness of regional eye health services is a barrier for cataract treatment [14].

Most people with cataract reported that they were unable to meet the costs of cataract surgery (n=234, 67%) and 52% reported that their family members also could not afford it. However, 189 (54.3%) were willing to spend money for treatment if they can afford it. It highlights the financial barrier as a main barrier for treatment seeking. Transportation services were not identified as a major barrier in this study. Yin et al. (2009) also did not find transport services and costs of treatment as barriers for cataract treatment. In the USA, Desai and Copeland (2013) described barriers for cataract surgeries as mainly socioeconomic, due to factors as insurance coverage, income, and accessing barriers such as transportation and service accessibility in contrast to the findings of this study. Boudville et al. (2012) described the cost of surgery as the main barrier for treatment. Abubakar et al. (2012) reported that one-third of people blind with cataract could not afford surgery [15]. Transportation may not have been identified as a main barrier in this study as the Mahara DS division is well connected by rail and road networks to the main hospitals in the district.

Three hundred twenty-seven (94%) persons with cataract believed that surgery can cure the cataract and 344 (99%) had believed that the quality of surgery is good. Yin et al. (2009) reported that concerns about the quality of local services appeared to be the principal barrier to cataract surgery in China [11].

Fear of cataract surgery, believing that “one eye is enough for me”, and fear of worsening current vision after a cataract surgery were also reported. Thirty-four percent of people thought that there was no need to intervene in the natural process of vision reduction. These misconceptions may be a reflection of the Buddhist culture of accepting physical changes as a normal process and belief of “karma”. Forty-eight percent thought that there would be suffering for their loved ones and family due to their surgery. Sixty-five percent of people thought that they may have to wait for long periods in waiting lists and experience long waiting times in hospital clinics. Boudville et al. (2012) described long waiting times as a barrier. Chandrashekhar et al. (2007) and Sapkota et al. (2004) identified the fear of the operation as a main barrier in studies conducted in India and Nepal respectively [8, 9].

The findings of this study are important for prioritizing cataract as a major public health problem associated with preventable disability. The importance of cataract cannot be overemphasized in Sri Lanka in the present context of rapidly ageing population. Findings of this study highlight the importance of cataract removing the barriers for its management which should be given due prominence in the formulation of public health policy in Sri Lanka at the earliest.

## Figures and Tables

**Table 1:** Sociodemographic profile of the participants by gender.

	Male (n=135)	Percentage (100%)	Female (n=335)	Percentage (100%)	Total (n=470)	Percentage (100%)
*Age category*						
60 – 64	50	37.0%	95	28.4%	145	30.9%
65 – 69	40	29.6%	117	34.9%	157	33.4%
70 – 74	16	11.9%	65	19.4%	81	17.2%
75 – 79	16	11.9%	33	9.9%	49	10.4%
80 > above	13	9.6%	25	7.5%	38	8.1%

*Civil status*						
Currently married	123	91.1%	233	69.4%	356	75.7%
Never married	6	4.4%	9	2.7%	15	3.2%
Divorced or widowed	6	8.9%	93	30.6%	99	24.3%

*Educational status*	-	-	-	-	-	
Primary or less	29	21.5%	56	16.7%	85	18.1%
Secondary to passed Ordinary level	82	60.7%	220	65.7%	302	64.2%
More than Ordinary level	24	17.8%	59	17.6%	83	17.7%

*Income (LKR)∗*						
Less than 5,000	45	33.3%	166	49.6%	211	44.9%
5,000 -19999	64	47.4%	110	32.8%	174	37.0%
More than 20,000	26	19.3%	59	17.6%	85	18.1%

*Financial Support*						
With some support	42	31.1%	129	38.5%	171	36.4%

*Expenses are covered by*	-	-	-	-	-	
Job	40	29.6%	46	13.7%	87	18.5%
Pension	29	21.5%	99	29.6%	128	27.2%
Children or relatives aids	59	43.7%	174	51.9%	233	49.6%
Other	7	5.2%	16	4.8%	22	4.7%

*Current occupation*						
Involved in a job	40	29.6%	45	13.4%	86	18.3%

*∗*per month

**Table 2:** Distribution and 95% CI of prevalence of different types of cataracts by gender.

		Males ( n= 135)				Females (n=335)		
	Frequency	%	95% C.I.		Frequency	%	95% C.I.	
Nuclear only	61	45.2%	37.0%	-53.6%	150	44.8%	39.%	-50.1%
Mixed	32	23.7%	17.3%	-31.5%	95	28.4%	23.%	-33.4%
Cortical only	1	0.7%	0.1%	- 4.1%	1	0.3%	0.1%	- 1.7%
Subcapsular only	3	2.2%	0.8%	- 6.3%	3	0.9%	0.3%	- 2.6%
Any cataract	107	79.3%	71.2%	85.2%	272	81.2%	76.7%	-85.0%

Treated (no cataract now)	10	7.4%	4.1%	- 13.1%	23	6.9%	4.6%	-10.1%
No cataract	28	20.7%	21.2%	- 36.3%	63	18.8%	21.%	-30.6%

**Table 3:** Level of knowledge about cataract and the cataract status among people with cataract.

Question	Yes		No		Don't know		
	N	%	N	%	n	%	Total
Did you know that you have cataract?	90	25.9%	258	74.1%	-	0.0%	348
Can cataract be cured?	218	62.6%	39	11.2%	91	26.1%	348
Is the definitive treatment for cataract is surgery?	184	52.7%	80	22.9%	85	24.4%	349
Do government hospitals provide treatment for cataract?	239	68.7%	46	13.2%	63	18.1%	348
Is it necessary to implant a lens in cataract surgery?	169	48.4%	79	22.6%	101	28.9%	349
Is it necessary to give general anaesthesia for cataract surgery?	104	30.1%	167	48.3%	75	21.7%	346
Can cataract surgery be done free of charge?	171	49.1%	113	32.5%	64	18.4%	348

**Table 4:** Financial, logistics, and transport barriers for cataract treatment.

Question	Yes		No		Total
	n	%	n	%	
Will you able to pay for the surgery?	115	33.0%	234	67.0%	349
Are you willing to pay for the surgery if you have money?	189	54.3%	160	46.0%	348
Are your family members able to pay for your surgery?	167	48.4%	179	51.9%	345
Is it possible for you to find transport to go to hospital?	311	89.4%	37	10.6%	348
Do you expect assistance from your family members/children/relatives for the surgery?	208	59.8%	140	40.2%	348
Will you able to pay for drugs?	280	80.7%	67	19.3%	347
Will your family members /relatives help you for the surgery?	268	77.0%	80	23.0%	348

**Table 5:** Social and other commitments affected for cataract treatment.

Question	Yes		No	
	n	%	n	%
Are you postponing your cataract surgery?	62	17.8%	286	82.2%
Are you postponing your surgery due to your busy employment schedule?	8	12.9%	54	87.1%
Are you postponing your surgery due to your family members busy work schedule?	20	32.3%	42	67.7%
Are you postponing your surgery due to your family commitments?	21	33.9%	41	66.1.0%
Are you postponing your surgery due to your social/ religious commitments?	3	4.8%	59	95.2%

**Table 6:** Attitudes and beliefs affecting the treatment seeking for cataract.

	Percentile *∗*		
Question	25th	50th	75th
I am afraid to undergo a cataract surgery	0	0	8
I am worried about the cost I have to incur for the cataract surgery	1	8	10
I am afraid that the operation will lead to lose my eye sight further more	0	0	2
I believe that I could manage my work with one eye	0	0	5
I am afraid that operation will make me away from my daily routine work for long time	0	0	6
I believe that my poor eye vision is natural process and no need to intervene for that	0	0	2
I am worried that my partner, children and relatives will have to suffer due to eye surgery	0	0	7
I am afraid that I will have to wait long periods of time in the waiting list to do the operation	0	5	10
I am afraid that I will have to hang around for a long time in hospitals and clinics to get the treatment	0	6	10
I believe that I am too old to undergo an eye surgery	0	0	2
I am afraid that I have to limit my daily work due cataract surgery	0	1	7

*∗*0= not at all and 10= very much.

## Data Availability

Corresponding author can be contacted for original data.
